# Hydrogen Sulfide Attenuates Aortic Remodeling in Aortic Dissection Associating with Moderated Inflammation and Oxidative Stress through a NO-Dependent Pathway

**DOI:** 10.3390/antiox10050682

**Published:** 2021-04-27

**Authors:** Hsin-Ying Lu, Hung-Lung Hsu, Chih-Han Li, Shao-Jung Li, Shing-Jong Lin, Chun-Ming Shih, Chun-Che Shih

**Affiliations:** 1 Division of Cardiovascular Surgery, Department of Surgery, Wan Fang Hospital, Taipei Medical University, Taipei 116, Taiwan; 108248@w.tmu.tw (H.-Y.L.); 101146@w.tmu.edu.tw (S.-J.L.); ccshih0603@tmu.edu.tw (C.-C.S.); 2 Institute of Clinical Medicine, National Yang Ming Chiao Tung University, Taipei 112, Taiwan; dennishsu90.y@nycu.edu.tw (H.-L.H.); 109317@w.tmu.tw (C.-H.L.); sjlin@vghtpe.gov.tw (S.-J.L.); 3 Taipei Heart Institute, Taipei Medical University, Taipei 110, Taiwan; 4 School of Medicine, National Yang Ming Chiao Tung University, Taipei 112, Taiwan; 5 Division of Cardiovascular Surgery, Far Eastern Memorial Hospital, Taipei 220, Taiwan; 6 Department of Surgery, School of Medicine, College of Medicine, Taipei Medical University, Taipei 110, Taiwan; 7 Division of Cardiology, Department of Internal Medicine, Taipei Medical University Hospital, Taipei 110, Taiwan; 8 Department of Internal Medicine, School of Medicine, College of Medicine, Taipei Medical University, Taipei 110, Taiwan

**Keywords:** hydrogen sulfide, aortic dissection, oxidative stress, inflammation, smooth muscle cell

## Abstract

Aortic dissection (AD) is a highly lethal vascular disease characterized by separation of the constituent layers of the aortic wall. An increasing body of research indicates that inflammatory response and oxidative stress are implicated in vascular remodeling, which plays a key role in the development of AD. Hydrogen sulfide (H_2_S) has been found to protect against various types of cardiovascular disease, including myocardial infarction, arthrosclerosis, and hypertension. However, research on the effect of H_2_S on AD is insufficient. This study therefore elucidated the effect of H_2_S on the development and progression of AD, and the potential mechanism involved. Using β-aminopropionitrile fumarate (BAPN) and angiotensin II (Ang-II)-induced AD animal models, the administration of NaHS (as H_2_S donor, 56 μmol/kg body weight/day) was found to retard the development of AD. Murine VSMCs (Movas) exposed to interleukin-6 (IL-6) (20 ng/mL) to induce phenotypic switch. Histological analyses indicated that H_2_S administration inhibited the accumulation of inflammatory cells in the aortic wall and the related expression of inflammatory genes. Additionally, H_2_S treatment elevated aortic superoxide dismutase (SOD) activity and ablated malonaldehyde (MDA) and nitric oxide (NO) levels. In mechanistic terms, H_2_S attenuated IL-6 induced a pathological VSMC phenotypical switch through NO modulation by N(G)-monomethyl-L-arginine acetate salt (L-NMMA) stimulation. H_2_S inhibits AD formation by decreasing the inflammatory response, and oxidative stress, and by positively participating in vascular remodeling. These findings suggest a role for H_2_S as a novel and promising therapeutic strategy to prevent AD development.

## 1. Introduction

Aortic dissection (AD) is defined as disruption of the medial layer provoked by intramural bleeding, resulting in separation of the aortic wall layers and subsequent formation of a true and a false lumen with or without communication [1]. It is a life-threatening disease involving arterial remodeling characterized by high levels of mortality and severe complications. The development of endovascular techniques has improved the diagnosis and treatment of AD [2], however, this involves certain limitations, and the mortality associated with aortic rupture remains high. Thus, developing effective and safe therapeutic strategies to prevent AD formation and progression is critical.

Reactive oxygen species (ROS) can regulate the cellular and extracellular components of the aortic wall in the dissecting of a thoracic aortic aneurysm [3]. Additionally, the involvement of inflammatory mechanisms in the process of arterial wall remodeling plays a key role in the development and progression of aortic dissection [4]. Activated T and B lymphocytes and macrophages have been reported inside the aortic wall around the vasa vasorum and at the edge of ruptured media [5]. These cells activate inflammatory reactions such as the secretion of various cytokines and chemokines implicated in vascular homeostatic imbalance. Furthermore, the key histopathologic feature of AD is medial degeneration, which is characterized by the depletion of vascular smooth muscle cells (VSMCs) and extracellular matrix degradation [6]. VSMCs play a valuable part in maintaining the structural integrity and function of vessels. Under pathologic stimulation, the quiescent contractile SMCs transform into an activate synthetic state, resulting in an increase in SMCs migration and extracellular matrix secretion [7]. This dedifferentiation process, known as a phenotype switch, is closely related to progressive aortic dilation and ultimately ruptures [8]. Therefore, maintenance of the VSMC contractile phenotype and suppression of vascular inflammation are essential in the prevention and treatment of vascular diseases.

Hydrogen sulfide (H_2_S) is considered the third gaseous signaling molecule alongside nitric oxide (NO) and carbon monoxide (CO) [9]. H_2_S is endogenously synthesized by three enzymes; cystathionine β-synthase (CBS), cystathionine-c-lyase (CSE), and 3-mercaptopyruvate sulfurtransferase (MST) [10]. Growing evidence suggests that H_2_S protects against various inflammations in injured tissues, such as those in the lung, heart, liver, and kidneys [11,12,13,14]. In vitro studies have confirmed this anti-inflammatory role through the modulation of NF-κB activity [15,16]. Furthermore, H_2_S primarily works as an antioxidant. Treatment with NaHS can inhibit vascular NADPH oxidase (NOX)-derived O2- production in vitro. Collectively, these findings suggest that H_2_S is capable of preventing inflammation and oxidative stress and thus has potential therapeutic applications. 

Numerous studies have reported that H_2_S has a range of biological effects on the cardiovascular system including hypertension [17], ischemia-reperfusion injury heart disease [18], atherosclerosis [19], and heart failure [20]. However, the effect of H_2_S on AD remains unknown. The aim of the present study was to determine whether exogenous H_2_S supplement attenuated AD in mice. 

## 2. Materials and Methods

### 2.1. Development of Aortic Dissection Model in Mice

Three-week-old male C57BL/6 mice were purchased from BioLASCO Co. Ltd. (Taipei, Taiwan) and fed a normal diet and 0.25% (*w*/*w*) β-aminopropionitrile (BAPN) (Sigma-Aldrich, St. Louis, MO, USA), which was dissolved in drinking water for 4 weeks. At 7 weeks old, osmotic minipumps (Model 1003D micro-osmotic pump; Alzet, Cupertino, CA, USA) administering either saline (control group, *n* = 17) or 1 μg/kg/min angiotensin II (Ang-II) (intervention group and model group) were implanted subcutaneously, and mice were euthanized 24 h after implantation. For the therapeutic intervention with NaHS, intraperitoneal injection of NaHS (56 μmol/kg) (*n* = 33) or vehicle (PBS) (*n* = 33) was started on day 7 from the beginning of BAPN administration. The animal studies were approved by the Laboratory Animal Center at Taipei Medical University (Taipei, Taiwan).

### 2.2. External Aortic Diameter Measurement

The external aortic diameter of the mice was measured in three transverse sections using image J software (National Institutes of Health), as described previously [21].The ascending aorta was defined as the proximal 2-mm section from the ostium of the innominate artery, the aortic arch as the section between the ostia of the left common carotid artery and left subclavian artery, and the descending aorta as the distal 2-mm section from the ostium of the left subclavian artery.

### 2.3. Histological and TUNEL Analysis

Resected aorta from mice were fixed in 4% formaldehyde overnight, dehydrated, paraffin-embedded, and cut into 4 μm thick sections. The tissue sections were then stained with hematoxylin and eosin (H&E) and elastin Verhoeff-van-Giessen (EVG) and examined under a light microscope. Qualitative evaluation of elastin integrity was performed by a blinded observer on digital images using semiquantitative grading, as described previously [22]. Elastin preservation was graded as follows: grade 1, nonelastin degradation, well organized elastin laminae; grade 2, elastic laminae with interruptions and breaks; grade 3, elastic laminae with multiple interruptions and breaks; and grade 4, severe elastin fragmentation or loss. A commercially available TUNEL apoptosis detection kit (Roche, Branchburg, NJ, USA) was employed to detect apoptosis in accordance with the manufacturer’s instructions. Positive TUNEL staining was observed under a fluorescence microscope and the percentage of apoptotic cells was calculated.

### 2.4. Immunohistochemistry

For immunohistochemistry, the sections were hydrated, heated for antigen retrieval, and treated with hydrogen peroxide to deactivate the endogenous peroxidase as per standard protocols. After blocking with 5% BSA, the sections were incubated overnight with primary antibodies to specifically detect CD3 (1:100, Abcam, Cat# ab5690), CD68 (1:100, Abcam, Cat# ab31630), MPO (1:1000, Abcam, Cat# ab208670), and IL-6 (1:50, GeneTex, Cat#GTX17623), along with the control IgG (1:100; Abcam, Cat# ab37415) at 4 °C. The sections were then incubated with a horseradish peroxidase-(HRP-) conjugated secondary antibody, washed with PBS, and stained with diaminobenzidine. Finally, the sections were counterstained with hematoxylin and viewed under a light microscope. 

### 2.5. Cell Culture and Treatments

MOVAS cells, murine VSMCs, were purchased from American Type Culture Collection (ATCC, Manassas, VA, USA) and maintained in Dulbecco modified Eagle medium containing 10% fetal bovine serum (FBS), 1% penicillin-streptomycin and 0.2 mg/mL G-418 (GIBCO) through incubation at 37 °C in a humidified atmosphere of 95% air/5% CO_2_. MOVAS cells were seeded in multiwell plates at a density of 1.0 × 10^4^ cells/cm^2^. At 100% confluence, cells were exposed to Interlukin-6 (IL-6) (20 ng/mL). A specific NO signal could be identified after incubation with N(G)-monomethyl-L-arginine acetate salt (L-NMMA, an inhibitor of NO synthesis), which facilitated detection of a specific electron spin resonance signal in the absence of NO.

### 2.6. Measurement of SOD Activity, Malondialdehyde Level, and NO Level

The aortic homogenates were used to perform the assays for oxidative stress. Total SOD activity was measured using specially designed kits (Invitrogen, Carlsbad, CA, USA) in accordance with the manufacturer’s instructions. The absorbance of the supernatant was measured at 450 nm, the result of which is expressed as U per mg protein. Lipid peroxidation was assessed by quantifying malonaldehyde (MDA) using a TBARS assay kit (Cayman Chemical Company, Ann Arbor, MI, USA) in accordance with manufacturer’s instructions. The absorbance of the supernatant was measured at 450 nm, the value of which is expressed as nmol of per mg protein. The NO concentration in aortic homogenates was determined using Griess reagent (1% sulfanilamide and 0.1% N-(1-naphthyl)ethylenediamine in 2% phosphoric acid). The absorbance was measured at 570 nm, the result of which is expressed as nmol per mg protein.

### 2.7. Quantitative Reverse Transcription-Polymerase Chain Reaction

Total RNA was extracted from a TRIzol reagent (Invitrogen) I accordance with the manufacturer’s instructions. The cDNA was synthesized using RevertAid First Strand cDNA Synthesis Kit (Thermo Scientific, Waltham, MA, USA), also in accordance with the manufacturer’s instructions. Reverse transcription-polymerase chain reaction (RT-PCR) analysis was performed using the SYBR Premix Ex Taq II (Takara) in ABI7500 Real-Time PCR Systems (Applied Biosystems, Foster City, CA, USA). The relative expression of each gene was normalized to the GAPDH gene and analyzed using the 2-∆∆CT method. The primer sequences were as follows: IL-6 forward: 5′-CCGGAGAGGAGACTTCACAG-3′, reverse: 5′-GGAAATTGGGGTAGGAAGGA-3′; IL-1β forward: 5′- GCACTACAGGCTCCGAGATGAAC-3′, reverse: 5′- TTGTCGTTGCTTGGTTCTCCTTGT-3′; TNF-α: 5′- GGAACTGGCAGAAGAGGCACTC-3′, reverse: 5′- GCAGGAATGAGAAGAGGCTGAGAC-3′; SM22α forward: 5′- GCAGTCCAAAATTGAGAAGA-3′, reverse: 5′- CTGTTGCTGCCCATTTGAAG-3′; Fibrillin-1 (FBN-1) forward: 5′- GATCAACGGCTACCCAAAAC-3′, reverse: 5′- GTTGGCTTCCATCTCAGACC-3′; Col3A1 forward: 5′- CCTCTGGTTCTCCTGGTCTG-3′, reverse: 5′- CCACCTTCACCCTTATCTCC-3′; and GAPDH forward: 5′- TCACCACCATGGAGAAGGC-3′, reverse: 5′- GCTAAGCAGTTGGTGGTGCA-3′.

### 2.8. Western Blotting

Protein was extracted from aortic tissues or Movas cells by using RIPA buffer plus protease inhibitors. SDS-PAGE was used to separate an equal amount of protein, which was then transferred to the PVDF membrane. After blocking, the membrane was incubated with diluted primary antibodies to detect cleaved Caspase-3 (1:1000, Cell signaling, Cat# 9664), SM22α (1:1000, proteintech, Cat# 10493-1-AP), p-STAT-3 (1:20,000, Abcam, Cat# ab76315), STAT-3 (1:1000, Abcam, Cat# ab68153), p-NF-κBp65 (1:1000, Cell signaling, Cat#3033), NF-κBp65 (1:000, proteintech, Cat# 10745-1-AP), and iNOS (1:500, Abcam, Cat# ab3523) overnight at 4 °C followed by incubation with a HRP-conjugated secondary antibody (Jackson Immuno Research, West Grove, PA, USA). Proteins were visualized using an ECL detection substrate (Millipore, Billerica, MA, USA) on UVP. GAPDH was detected as a loading control.

### 2.9. Detection of Reactive Oxygen Species

Cells were incubated with 5 μM DCFH-DA (Invitrogen) for 30 min at 37 °C. The reaction oxygen species (ROS) level was monitored at 488 nm excitation and 515 nm emission.

### 2.10. Cell Proliferation Assay

Cell proliferation was determined using a WST-1 assay (Roach). Movas cells were seeded in 96-well plates (5000 cells/well). After the treatment, 10 μL WST-1 was added and the cells were incubated for another 2 h at 37 °C in the incubator. The absorbance was monitored at 450 nm.

### 2.11. Statistical Analysis

Data are presented as mean ± SD. Experiments were repeated at least three times. Statistical analysis was performed using GraphPad Prism (version 5.0, GraphPad Software). Multigroup comparisons were performed using one way ANOVA with the post hoc Tukey test for equal variance or Dunnett T3 test for unequal variance. Kaplan–Meier survival curves were plotted to examine mouse survival rates, and the differences were analyzed with the log-rank (Mantel–Cox) test. For all statistical analyses, 2-tailed probability values were used. Statistical significance was defined as *p* < 0.05.

## 3. Results

### 3.1. H_2_S Suppresses the Development of AD in Animal Models

**Figure 1:**
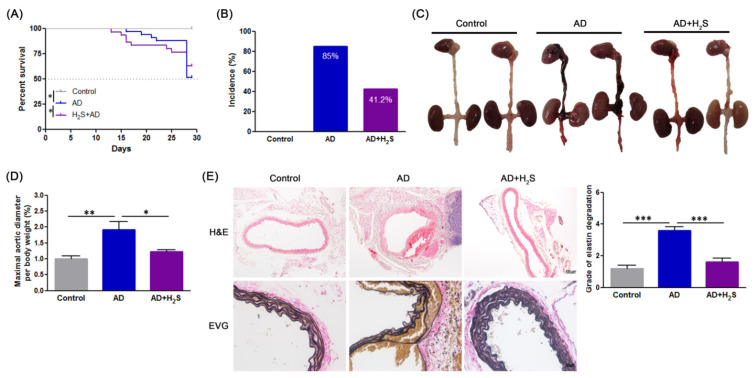
H_2_S ameliorates the etiopathology of BAPN/Ang-II-induced AD in mice. (**A**) Survival curves in indicated groups. (**B**) Incidence of AD. (**C**) Representative morphologies of aortas. (**D**) Maximal aortic diameter (normalized to body weight). (**E**) Representative HE and EVG staining in aorta, and quantification of elastin degradation in aortic wall (*n* = 5 per group). The data are expressed as mean ± SD. Statistical significance: * *p* < 0.05, ** *p* < 0.01, *** *p* < 0.001.

As indicated in Figure 1A, the survival curve for H_2_S-treated mice were significantly better (log-rank *p* < 0.05) than those for AD mice. 85% (28/33) of the BAPN/Ang-II infused mice developed AD, whereas only 42% (14/33) of the H_2_S-treated of BAPN/Ang-II infused mice developed AD (Figure 1B). Additionally, treating AD mice with H2S preserved aortic structure (Figure 1C) and maximal aortic diameters (Figure 1D). Furthermore, the histological examination of aortas with H&E and EVG (Figure 1E) indicated false lumen formation, severe degeneration, and collapse of the elastic lamina. In contrast, the media and adventitial thickness, and the elastic lamina remained intact in H_2_S-treated mice. These results suggested that H_2_S attenuated AD evolution.

### 3.2. H_2_S Mitigates Cell Death of Aortic Dissection

**Figure 2:**
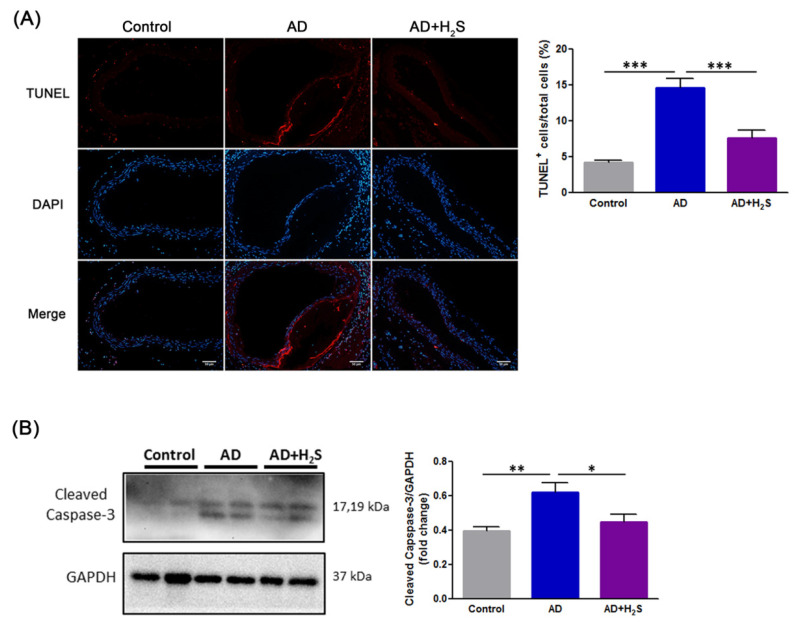
Treatment with H_2_S suppresses cell death in AD mice. (**A**) Representative photographs of TUNEL staining. Scale bars, 50 μm. Apoptotic cells are shown in red and cell nuclei in blue. Quantification of TUNEL positive cells in the region of the aortas (*n* = 5 per group). The data are presented as means ± SD. Statistical significance: *** *p* < 0.001. (**B**) Representative Western blot analysis of cleaved capspase-3 expressions in aortic tissues of each treatment group (*n* = 5 per group). GAPDH was used as a loading control. Quantification of protein levels are presented as means ± SD. Statistical significance: * *p* < 0.05, ** *p* < 0.01, *** *p* < 0.001.

TUNEL staining was performed to detect apoptosis in aortic samples from mice. The results demonstrated that the AD group exhibited significantly increased cell apoptosis; however, H_2_S treatment attenuated the apoptotic cells (Figure 2A). Western blotting indicated that the AD group exhibited significantly higher cleaved caspase-3 levels than the control group. Notably, H_2_S administration reduced the expressions of cleaved caspase-3 (Figure 2B). The data indicated that H_2_S declined cell death in AD.

### 3.3. H_2_S Inhibits the Accumulation of Inflammatory Cells and Cytokine Profile Expression and Oxidative Stress in AD Mice Aorta

**Figure 3:**
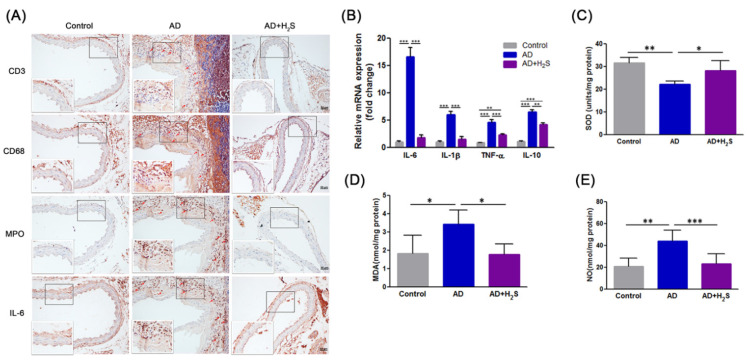
Administration of H_2_S reduced inflammatory response and oxidative stress in AD mice. (**A**) Representative immunohistochemical staining for T cells (CD3), macrophages (CD68), neutrophils (MPO), and IL-6 in transverse cryosections of aortas from mice (*n* = 3 per group). Scale bar, 20 μm. (**B**) RT–qPCR analysis of IL-6, IL-1β, TNF-α, and IL-10 mRNA levels in the aorta (*n* = 5 per group). (**C**) SOD activity, (**D**) MDA level, and (**E**) NO level were measured using aortic homogenates in each treatment group (*n* = 5 pre group). The data are expressed as mean ± SD. Statistical significance: * *p* < 0.05, ** *p* < 0.01, *** *p* < 0.001.

Immunohistochemical analysis was performed on the aorta of mice to evaluate the extent of inflammatory cell infiltration. As illustrated in Figure 3A, aortas from the AD group contained abundant CD3, CD68, and MPO positive cells and IL-6 expression. These were located primarily in the tunica adventitia, with fewer inflammatory cells scattered throughout the aortic tissues of H_2_S-administered mice. We also examined the effects of H_2_S treatment on the expression of proinflammatory cytokines in the aorta; these cytokines are known to be involved in AD development. The analysis of aortic specimens revealed that mRNA expression levels of IL-6, IL-1β, TNF-α, and IL-10 were markedly higher in the AD group. By contrast, the upregulation of these factors was significantly suppressed in the H_2_S group (Figure 3B).

Oxidative stress also contributes to the development and progression of AD. To evaluate whether H_2_S exerts an antioxidant effect in the aorta during AD formation, levels of SOD, MDA, and NO were measured. As illustrated in Figure 3C, the AD group exhibited lower aortic SOD activity than the control group, although treatment with H_2_S significantly restored SOD activity. By contrast, the AD group exhibited a significant increase in the levels of MDA compared with the control group, which was attenuated by H_2_S administration (Figure 3D). Moreover, ROS-mediated NO production affected aortic dissection. The levels of NO were therefore detected using the Griess method. The production of NO increased in the AD group compared with the control group. However, the levels of NO tended to be lower in the H_2_S group (Figure 3E).

These findings revealed that H_2_S administration ameliorated inflammatory cells infiltration into aortic tissue; the expression of inflammatory mediators; and oxidative stress preceding AD formation. H_2_S might therefore protect mice from AD development.

### 3.4. H_2_S Reverses the Expression of Synthetic Phenotypic Markers in AD Aorta

**Figure 4:**
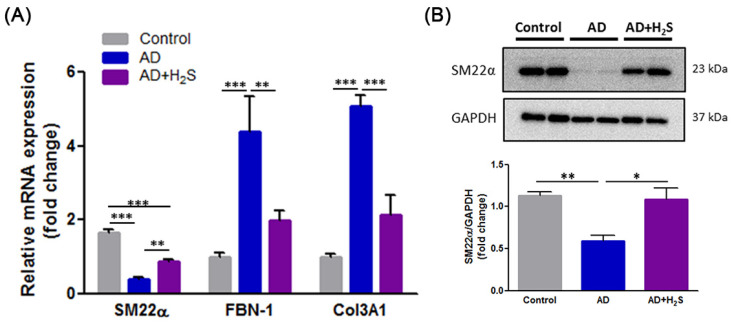
Protective effect of H_2_S maintains VSMC contractile phenotype. (**A**) The expression of SM22α, FBN-1, and Col3A1 mRNA were examined using RT-qPCR in the aorta of each treatment group (*n* = 5 per group). The data are expressed as mean ± SD. Statistical significance: ** *p* < 0.01, *** *p* < 0.001. (**B**) Western blot analysis of SM22α expression in aortic tissue extracts from different groups of mice. GAPDH was used as a loading control. Quantification of protein levels are presented as means ± SD. Statistical significance: * *p* < 0.05, ** *p* < 0.01.

VSMCs switch from a contractile to a synthetic phenotype that has been attributed to vascular malfunction under pathological conditions [8]. mRNA sequencing was thus performed to discern differential gene expressions in aortic tissues from experimental mice. The results revealed that the SM22α gene was downregulated whereas the FBN-1 and Col3A1 genes were upregulated. However, H_2_S treatment reversed these alternate gene expressions (Figure 4A). Western blotting further revealed that H_2_S administration countered the undesirable changes in SM22α (Figure 4B). These results demonstrated that H_2_S has the ability to maintain the VSMC contractile phenotype. Our data thus suggested that H_2_S treatment prevents the VSMC phenotypic switch in AD.

### 3.5. H_2_S Ameliorates Oxidative Stress and Phenotypic Switch in IL-6-Indcued VSMCs

**Figure 5:**
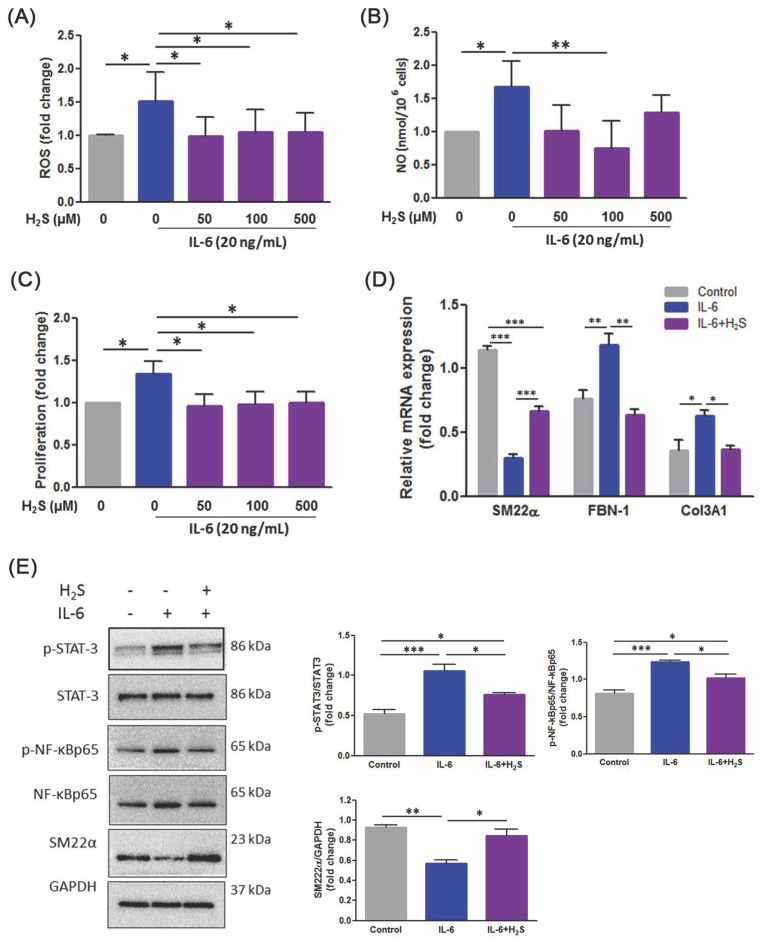
H_2_S effectively protects against IL-6 induced production of ROS and NO, and phenotypic switch in VSMCs. (**A**) The intracellular ROS level was detected using the fluorescent probe DCF-DA. (**B**) Changes in the NO level were measured using a Griess assay. (**C**) Cell proliferation was examined using a WST-1 assay. (**D**) RT–qPCR analysis of SM22α, Fibrillin-1 (FBN-1), and Col3A1 mRNA in VSMCs. (**E**) Western blotting was performed to detect the expression of p-STAT3/STAT3, p-NFkBp65/NF-kBp65, and SM22α. GAPDH was used as a loading control. Each result represents three separate experiments with qualitatively similar results. The data are expressed as mean ± SD. Statistical significance: * *p* < 0.05, ** *p* < 0.01, *** *p* < 0.001.

Previous research has indicated that IL-6 induced the degradation of contractile proteins by upregulating autophagy in thoracic AD VSMCs [23]. We therefore tested the effects of H_2_S using IL-6 induced mouse VSMCs. The results indicated that treatment with H_2_S could eradicate IL-6-induced ROS (Figure 5A) and NO production (Figure 5B). We then measured the effects of H_2_S on VSMC proliferation following IL-6 stimulation. As illustrated in Figure 5C, H2S significantly inhibited IL-6-enhanced cell viability of VSMC. Additionally, H_2_S altered the mRNA level of SM22α, FBN-1, and Col3A1 by IL-6-stimulated VSMCs (Figure 5D). Finally, Western blotting indicated that VSMC treatment with IL-6 triggered the upregulation of p-STAT-3 and P-NF-kBp65 and the downregulation of SM22α, which induced the differentiation of VSMCs, was altered by H_2_S administration (Figure 5E). 

The results demonstrated that H2S attenuated IL-6-triggered phenotypic switching through modulation of oxidative stress and inflammation in VSMCs.

### 3.6. H_2_S Modulates IL-6-Induced Phenotype Switch of VSMCs through the ROS-NO-iNOS Pathway

**Figure 6:**
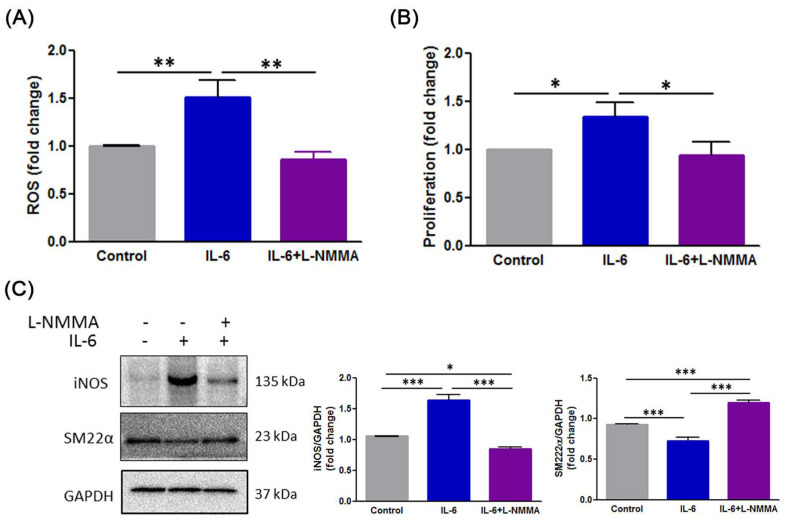
ROS-NO-iNOS axis governs IL-6-induced phenotypic switch in VSMCs. (**A**) The intracellular ROS level was measured using the fluorescent probe DCF-DA. (**B**) Cell proliferation was examined using a WST-1 assay. (**C**) Western blotting was performed to assess the expression of iNOS and SM22α. GAPDH was used as a loading control. Each result represents three separate experiments with qualitatively similar results. The data are expressed as mean ± SD. Statistical significance: * *p* < 0.05, ** *p* < 0.01, *** *p* < 0.001.

Based on observations from the present study, we predicted that the benefits of H_2_S influence NO action to ameliorate vascular remodeling. Therefore, to further clarify whether IL-6-induced the phenotypic switch of VSMC implicated in NO, an NOS inhibitor, L-NMMA, was employed. As illustrated in Figure 6A, L-NMMA significantly downregulated IL-6 induced ROS production in VSMC. In addition, L-NMMA inhibited IL-6 induced cell proliferation (Figure 6B). L-NMMA-treated cells also exhibited a change in the expression of iNOS and SM22α following IL-6 stimulation (Figure 6C). Our results suggest that H_2_S alleviated the phenotypic switch of VSMC during IL-6-induced through the regulation of the ROS-NO-iNOS axis.

## 4. Discussion

**Figure 7:**
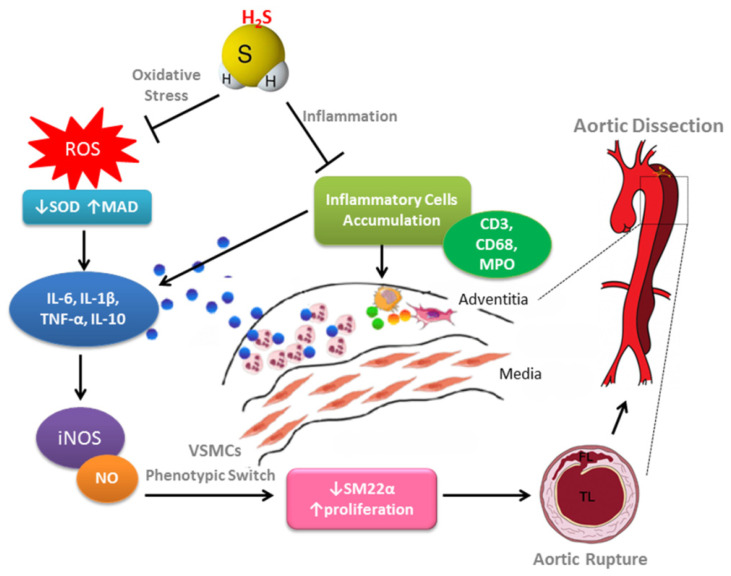
The proposed mechanism of H_2_S protects against AD formation. Exogenous H_2_S plays a protective role in vascular remodeling that involves the regulation of oxidative stress, inflammation, NO bioavailability, and cell death. Additionally, the phenotypic switch of VSMCs is a critical characteristic for AD progression and development. H_2_S inhibits IL-6-induced phenotypic switch of VSMCs through the ROS-NO-iNOS pathway.

This is the first study to explore whether H_2_S is capable of alleviating AD development in mice. The proposed mechanism of H_2_S that protects against from AD was elucidated in Figure 7.

Patients with AD exhibited an increase in inflammatory biomarkers including peripheral blood natural killer cells, B cells, regulatory T cells, C-reactive proteins, and cytokines such as IL-6 and IL-8, TNF-α, and C-C motif chemokine ligand 2 (CCL2) [4]. The granulocyte–macrophage colony-stimulating factor and macrophage infiltration into the aortic lesion site is required for AD formation [24]. Additionally, neutrophil infiltration derived by a C-X-C motif chemokine ligand 1 (CXCL1) has also been reported to induce progression from aortic dissection to aortic rupture in mice fed with BAPN and infused with Ang-II [25]. In the present study, H_2_S intervention alleviated vascular inflammation by reducing the inflammatory infiltration of macrophages, CD3+ T cells, and neutrophils, which provides a reasonable explanation for the lower incidence of AD in mice.

Oxidative stress is one of the main determinants of pathological remodeling of the arterial wall [26]. A study targeting AD patients documented an increase in MDA expression and a decrease in SOD expression of thoracic aortic tissues [27]. Moreover, oxidative stress was reported to aggravate the degree of vascular stiffness by regulating the production of elastic fibers, thereby increasing vascular wall shear stress, which led to AD [28]. In our study, the disruption of elastin fiber significantly increased in the AD group, whereas H_2_S reduced this value, indicating that H_2_S has the ability to improve structural integrity by antioxidative stress.

Another important finding in the present study was that H_2_S inhibited the phenotypic switch of VSMC from the contractile to synthetic type, thereby maintaining quantitative and functional homeostasis. The normal aortic media comprises a multitude of regularly arranged VSMCs and an extracellular matrix enriched with elastic fibers. Thus, SMCs clearly serve an essential role in the progression of AD, which may be involved in the structure and function of aortic tissues [29]. In patients with AD, a significant increase in the number and ratio of synthetic VSMCs was observed, resulting in decreased aortic elasticity and rupture of the vessel wall [30]. The quantity and phenotype changes of VSMCs in aortic media were part of an important pathological process of vascular remodeling, resulting in AD.

Sustained production of NO through iNOS is a response to various agents, including proinflammatory cytokines [31]. In VSMCs, iNOS is a predominant enzyme that promotes NO generation, and it is regarded as a main meditator of NO-dependent S-nitrosylation, and plays a potentially detrimental role in cardiovascular diseases [32,33,34]. The ability of adenosine to increase basal and cytokine- or lipopolysaccharide (LPS)-stimulated NO release in VSMCs is documented [35,36]. The increased release of NO derived from iNOS is involved in the development of diabetic vascular complications [37,38]. NO produced by iNOS plays a pivotal role in the pathogenesis of inflammation and aneurysm. iNOS inhibition has yielded positive outcomes in limiting aneurysm expansion in an elastase-induced aneurysm model and in the prevention of cerebral aneurysm formation in a hypertensive rat model [39]. In the present study, we investigated that H_2_S attenuated the IL-6-stimulated ROS production and NO-releasing derived from iNOS is involved in the VSMC phenotypic switch. In accordance with these findings, we found that the mechanistic basis for the protective role of H_2_S for VSMC phenotype in AD formation, at least in part, is through the ROS-NO-iNOS signaling pathway. 

## 5. Conclusions

The present study provides valuable insights into the potential role of H_2_S in preventing AD formation by attenuating the inflammatory response and oxidative status involved in vascular wall remodeling. Based on these findings, H_2_S for AD intervention might be of significant benefit.

## Data Availability

Not applicable.

## References

[1] Erbel R., Aboyans V., Boileau C., Bossone E., Bartolomeo R.D., Eggebrecht H., Evangelista A., Falk V., Frank H., Gaemperli O. (2014). 2014 ESC Guidelines on the diagnosis and treatment of aortic diseases: Document covering acute and chronic aortic diseases of the thoracic and abdominal aorta of the adult. The Task Force for the Diagnosis and Treatment of Aortic Diseases of the European Society of Cardiology (ESC). Eur. Heart J..

[2] Chen L.W., Wu X.J., Lu L., Zhang G.C., Yang G.F., Yang Z.W., Dong Y., Cao H., Chen Q. (2011). Total arch repair for acute type A aortic dissection with 2 modified techniques: Open single-branched stent graft placement and reinforcement of the dissected arch vessel stump with stent graft. Circulation.

[3] Maiellaro-Rafferty K., Weiss D., Joseph G., Wan W., Gleason R.L., Taylor W.R. (2011). Catalase overexpression in aortic smooth muscle prevents pathological mechanical changes underlying abdominal aortic aneurysm formation. Am. J. Physiol. Heart Circ. Physiol..

[4] del Porto F., Proietta M., Tritapepe L., Miraldi F., Koverech A., Cardelli P., Tabacco F., de Santis V., Vecchione A., Mitterhofer A.P. (2010). Inflammation and immune response in acute aortic dissection. Ann. Med..

[5] He R., Guo D.C., Estrera A.L., Safi H.J., Huynh T.T., Yin Z., Cao S.N., Lin J., Kurian T., Buja L.M. (2006). Characterization of the inflammatory and apoptotic cells in the aortas of patients with ascending thoracic aortic aneurysms and dissections. J. Thorac. Cardiovasc. Surg..

[6] Wu D., Shen Y.H., Russell L., Coselli J.S., LeMaire S.A. (2013). Molecular mechanisms of thoracic aortic dissection. J. Surg. Res..

[7] Wang L., Zhang J., Fu W., Guo D., Jiang J., Wang Y. (2012). Association of smooth muscle cell phenotypes with extracellular matrix disorders in thoracic aortic dissection. J. Vasc. Surg..

[8] Clement M., Chappell J., Raffort J., Lareyre F., Vandestienne M., Taylor A.L., Finigan A., Harrison J., Bennett M.R., Bruneval P. (2019). Vascular Smooth Muscle Cell Plasticity and Autophagy in Dissecting Aortic Aneurysms. Arter. Thromb. Vasc. Biol..

[9] Szabo C. (2016). Gasotransmitters in cancer: From pathophysiology to experimental therapy. Nat. Rev. Drug. Discov..

[10] Kamoun P. (2004). Endogenous production of hydrogen sulfide in mammals. Amino Acids.

[11] Li L., Hsu A., Moore P.K. (2009). Actions and interactions of nitric oxide, carbon monoxide and hydrogen sulphide in the cardiovascular system and in inflammation—A tale of three gases!. Pharmacol. Ther..

[12] Shimada S., Fukai M., Wakayama K., Ishikawa T., Kobayashi N., Kimura T., Yamashita K., Kamiyama T., Shimamura T., Taketomi A. (2015). Hydrogen sulfide augments survival signals in warm ischemia and reperfusion of the mouse liver. Surg. Today.

[13] Kasinath B.S. (2014). Hydrogen sulfide to the rescue in obstructive kidney injury. Kidney Int..

[14] Tokuda K., Kida K., Marutani E., Crimi E., Bougaki M., Khatri A., Kimura H., Ichinose F. (2012). Inhaled hydrogen sulfide prevents endotoxin-induced systemic inflammation and improves survival by altering sulfide metabolism in mice. Antioxid. Redox Signal..

[15] Yang C., Yang Z., Zhang M., Dong Q., Wang X., Lan A., Zeng F., Chen P., Wang C., Feng J. (2011). Hydrogen sulfide protects against chemical hypoxia-induced cytotoxicity and inflammation in HaCaT cells through inhibition of ROS/NF-kappaB/COX-2 pathway. PLoS ONE.

[16] Du J., Huang Y., Yan H., Zhang Q., Zhao M., Zhu M., Liu J., Chen S.X., Bu D., Tang C. (2014). Hydrogen sulfide suppresses oxidized low-density lipoprotein (ox-LDL)-stimulated monocyte chemoattractant protein 1 generation from macrophages via the nuclear factor kappaB (NF-kappaB) pathway. J. Biol. Chem..

[17] Xiao L., Dong J.H., Teng X., Jin S., Xue H.M., Liu S.Y., Guo Q., Shen W., Ni X.C., Wu Y.M. (2018). Hydrogen sulfide improves endothelial dysfunction in hypertension by activating peroxisome proliferator-activated receptor delta/endothelial nitric oxide synthase signaling. J. Hypertens..

[18] Elrod J.W., Calvert J.W., Morrison J., Doeller J.E., Kraus D.W., Tao L., Jiao X., Scalia R., Kiss L., Szabo C. (2007). Hydrogen sulfide attenuates myocardial ischemia-reperfusion injury by preservation of mitochondrial function. Proc. Natl. Acad. Sci. USA.

[19] Wang Y., Zhao X., Jin H., Wei H., Li W., Bu D., Tang X., Ren Y., Tang C., Du J. (2009). Role of hydrogen sulfide in the development of atherosclerotic lesions in apolipoprotein E knockout mice. Arter. Thromb. Vasc. Biol..

[20] Kondo K., Bhushan S., King A.L., Prabhu S.D., Hamid T., Koenig S., Murohara T., Predmore B.L., Gojon G., Gojon G. (2013). H(2)S protects against pressure overload-induced heart failure via upregulation of endothelial nitric oxide synthase. Circulation.

[21] Zhang L., Zhou J., Jing Z., Xiao Y., Sun Y., Wu Y., Sun H. (2018). Glucocorticoids Regulate the Vascular Remodeling of Aortic Dissection Via the p38 MAPK-HSP27 Pathway Mediated by Soluble TNF-RII. EBioMedicine.

[22] Middleton R.K., Lloyd G.M., Bown M.J., Cooper N.J., London N.J., Sayers R.D. (2007). The pro-inflammatory and chemotactic cytokine microenvironment of the abdominal aortic aneurysm wall: A protein array study. J. Vasc. Surg..

[23] An Z., Qiao F., Lu Q., Ma Y., Liu Y., Lu F., Xu Z. (2017). Interleukin-6 downregulated vascular smooth muscle cell contractile proteins via ATG4B-mediated autophagy in thoracic aortic dissection. Heart Vessel..

[24] Son B.K., Sawaki D., Tomida S., Fujita D., Aizawa K., Aoki H., Akishita M., Manabe I., Komuro I., Friedman S.L. (2015). Granulocyte macrophage colony-stimulating factor is required for aortic dissection/intramural haematoma. Nat. Commun..

[25] Anzai A., Shimoda M., Endo J., Kohno T., Katsumata Y., Matsuhashi T., Yamamoto T., Ito K., Yan X., Shirakawa K. (2015). Adventitial CXCL1/G-CSF expression in response to acute aortic dissection triggers local neutrophil recruitment and activation leading to aortic rupture. Circ. Res..

[26] Martin-Ventura J.L., Madrigal-Matute J., Martinez-Pinna R., Ramos-Mozo P., Blanco-Colio L.M., Moreno J.A., Tarin C., Burillo E., Fernandez-Garcia C.E., Egido J. (2012). Erythrocytes, leukocytes and platelets as a source of oxidative stress in chronic vascular diseases: Detoxifying mechanisms and potential therapeutic options. Thromb. Haemost..

[27] Liao M., Liu Z., Bao J., Zhao Z., Hu J., Feng X., Feng R., Lu Q., Mei Z., Liu Y. (2008). A proteomic study of the aortic media in human thoracic aortic dissection: Implication for oxidative stress. J. Thorac. Cardiovasc. Surg..

[28] Angouras D., Sokolis D.P., Dosios T., Kostomitsopoulos N., Boudoulas H., Skalkeas G., Karayannacos P.E. (2000). Effect of impaired vasa vasorum flow on the structure and mechanics of the thoracic aorta: Implications for the pathogenesis of aortic dissection. Eur. J. Cardiothorac Surg..

[29] Wei X., Sun Y., Wu Y., Zhu J., Gao B., Yan H., Zhao Z., Zhou J., Jing Z. (2017). Downregulation of Talin-1 expression associates with increased proliferation and migration of vascular smooth muscle cells in aortic dissection. BMC Cardiovasc. Disord..

[30] Cai Y.L., Wang Z.W. (2017). The expression and significance of IL-6, IFN-gamma, SM22alpha, and MMP-2 in rat model of aortic dissection. Eur. Rev. Med. Pharmacol. Sci..

[31] Dinerman J.L., Lowenstein C.J., Snyder S.H. (1993). Molecular mechanisms of nitric oxide regulation. Potential relevance to cardiovascular disease. Circ. Res..

[32] Schackelford R.E., Misra U.K., Florine-Casteel K., Thai S.F., Pizzo S.V., Adams D.O. (1995). Oxidized low density lipoprotein suppresses activation of NF kappa B in macrophages via a pertussis toxin-sensitive signaling mechanism. J. Biol. Chem..

[33] Kibbe M., Billiar T., Tzeng E. (1999). Inducible nitric oxide synthase and vascular injury. Cardiovasc. Res..

[34] Sha Y., Marshall H.E. (2012). S-nitrosylation in the regulation of gene transcription. Biochim. Biophys. Acta.

[35] Seo H.G., Fujii J., Asahi M., Okado A., Fujiwara N., Taniguchi N. (1997). Roles of purine nucleotides and adenosine in enhancing NOS II gene expression in interleukin-1 beta-stimulated rat vascular smooth muscle cells. Free Radic Res..

[36] Ikeda U., Kurosaki K., Ohya K., Shimada K. (1997). Adenosine stimulates nitric oxide synthesis in vascular smooth muscle cells. Cardiovasc. Res..

[37] Chen H., Brahmbhatt S., Gupta A., Sharma A.C. (2005). Duration of streptozotocin-induced diabetes differentially affects p38-mitogen-activated protein kinase (MAPK) phosphorylation in renal and vascular dysfunction. Cardiovasc. Diabetol..

[38] Nagareddy P.R., Xia Z., McNeill J.H., MacLeod K.M. (2005). Increased expression of iNOS is associated with endothelial dysfunction and impaired pressor responsiveness in streptozotocin-induced diabetes. Am. J. Physiol. Heart Circ. Physiol..

[39] Johanning J.M., Franklin D.P., Han D.C., Carey D.J., Elmore J.R. (2001). Inhibition of inducible nitric oxide synthase limits nitric oxide production and experimental aneurysm expansion. J. Vasc. Surg..

